# Associations between accelerometer-measured physical activity and body fatness in school-aged children

**DOI:** 10.1186/s12199-017-0629-4

**Published:** 2017-04-28

**Authors:** Aleš Gába, Josef Mitáš, Lukáš Jakubec

**Affiliations:** Faculty of Physical Culture, Palacký University Olomouc, třída Míru 117, Olomouc, 771 11 Czech Republic

**Keywords:** Body composition, Body fat distribution, Body mass index, Pediatric obesity, Moderate-to-vigorous physical activity

## Abstract

**Background:**

The main aim of the study was to examine the cross-sectional associations between objectively measured physical activity (PA) and body fatness in 7–12-year-old children.

**Methods:**

We performed an analysis of 365 children (209 girls). Participant recruitment was performed in eight randomly selected elementary schools in cities and towns with various numbers of inhabitants. The body composition analysis was performed according to a multi-frequency bioelectrical impedance analysis; PA was monitored using an accelerometer.

**Results:**

In terms of the overall PA, boys were more active than girls. No significant associations (unadjusted and adjusted models) were found between light PA and all body fatness indicators in either sex. Moderate-to-vigorous PA was significantly negatively associated with all body fatness indicators only in girls. These associations strengthened after adjustment for age, height and sedentary time (β ranging from –0.49 to –0.36, *P* ≤ 0.01). In contrast, vigorous PA was strongly negatively associated with body fatness indicators only in boys. In the fully adjusted model the significant negative associations were found for fat mass percentage (β = –0.15, *P* = 0.048) and fat mass index (β = –0.15, *P* = 0.040).

**Conclusions:**

The present study suggests that increasing sex-specific PA of different intensities may be an appropriate approach for decreasing body fatness in children. Longitudinal studies are needed to verify these associations.

## Background

During the last three decades, the global prevalence of obesity has doubled [1]. Although there is evidence for a slower increase in the prevalence of overweight and obesity in selected population groups in developed countries [2, 3], the number of individuals with overweight and obesity is rapidly increasing in middle- and low-income countries, particularly in urban settings [4]. It is estimated that more than 90 million of children aged 5–17 years will be obese by 2025 if effective policy interventions do not change the current trends [5].

For a long time, the Czech Republic has been among the European countries with a high prevalence of overweight and obesity in all age categories, and it is estimated that 18% of girls and 22.3% of boys younger than 20 years are overweight and obese [6]. A continuous increase in the prevalence of overweight and obesity in Czech children and adolescents was observed between 1951 and 2001 [7, 8]; in the last decade, this trend was confirmed by the results of the Health Behavior in School-aged Children study [9].

Scientific evidence clearly demonstrates that overweight and obesity in childhood and adolescence have adverse consequences on premature mortality and physical morbidity in adulthood [10]. For this reason, limiting or even stopping the global increase in the prevalence of childhood obesity is considered one of the main challenges of today’s society. Physical activity (PA) appears to be an important component for preventing obesity [11, 12]. Many authorities agree that children and adolescents should perform at least 60 min∙day^−1^ of moderate-to-vigorous physical activity (MVPA) [13–16]. Unfortunately 80% of youths do not meet this recommendation worldwide [17] and the transition from childhood to adolescence is accompanied by a significant decrease in habitual PA [18, 19].

Regarding the fact that PA is one of the main modifiable obesogenic behaviors, the associations between PA and body fatness have been widely studied. Unfortunately, comparing the results of different studies is challenging, especially due to inconsistences in the methods used for the assessment of body fatness and PA level. Several studies used simple anthropometric proxies for body fatness as outcomes (e.g. BMI, BMI z-score) and/or self-reported methods for PA assessment rather than more precise measures. The accuracy of self-reports remains still questionable; therefore, precise instruments must be used to gain accurate measures of associations between PA and total and regional body fatness.

Pooling the results from different studies support the hypothesis of an overall protective effect of PA on body fatness in children and adolescents [20, 21]. With respect to sexual dimorphism in total adiposity and its regional distribution during childhood [22], sex is one of the main factors influencing the strength of associations. Although a majority of cross-sectional [23, 24] and prospective studies [25] confirmed a sex-specific association between PA and body fatness, there are also studies that report no differences between girls and boys [26, 27]. Similarly, PA components (e.g., intensity, frequency, energy expenditure) have a significant influence on the associations between PA and body fatness. Scientific evidence shows that higher intensity PA is more protective against excess body fatness than total activity [21, 24–27] and this relationship seems to be independent of sedentary time [28]. Understanding the role of the above-mentioned factors may be essential for primary and secondary prevention of childhood obesity and its comorbidities. Therefore, the main purpose of this study was to examine the cross-sectional associations between objectively measured PA with various intensities and body fatness, represented as fat mass (FM), fat mass percentage (FM%), fat mass index (FMI) and visceral adipose tissue (VAT), separately in a sample of Czech girls and boys aged from 7 to 12 years.

## Methods

### Participants

We randomly selected 24 elementary schools in the eastern part of the Czech Republic (Moravia region) to investigate the body composition (BC) and PA in grades 2 to 5 pupils. The selection did not include sports schools (sports academies) or schools for pupils with special educational needs. A total of 8 elementary schools consented to the research (33% response rate). To ensure a representative sample, the schools were selected in cities and towns of various numbers of inhabitants [≥100 000 (*N* = 4), ≥25 000 (*N* = 2), ≥5 000 (*N* = 1) and <5 000 (*N* = 1)].

The main inclusion criteria were age (7–12 years) and a good health condition. Basic information about the objectives and content of the research study were presented to the parents using an information booklet. The parents were also given a telephone number to inquire about any additional information or to clarify the objective and extent of the study. In this way, we addressed ~1 600 parents, and 620 of them agreed for their children (*N* = 632) to participate in the research (~39% response rate) and provided written consent. If children reported medical complications that could influence the results of the measurement of BC and PA, a full examination was performed for ethical reasons; however, the results were not included in the final analysis. The basic sample description is shown in Table 1. To eliminate seasonal fluctuations in BC and PA, the research study was performed in two relatively similar seasons (spring and autumn).

**Table 1:** Descriptive characteristics of the participants, weight and physical activity status

	Girls (*N* = 209)					Boys (*N* = 156)				
	Mean	SD	95% CI			Mean	SD	95% CI		
Age and anthropometrics										
Age, years	9.8	1.3	9.6	to	10.0	9.9	1.2	9.7	to	10.0
Body height, cm	142.5	8.9	141.3	to	143.7	143.8	9.4	142.3	to	145.2
Body weight, kg	35.8	8.8	34.6	to	37.0	37.4	9.2	36.0	to	38.9
BMI, kg · m^−2^	17.4	2.8	17.0	to	17.8	17.9	2.8	17.4	to	18.3
Weight status, *N* (% of *N*)^a^										
Underweight	9 (4)					8 (5)				
Normal weight	152 (73)					113 (73)				
Overweight	26 (12)					25 (16)				
Obesity	22 (11)					10 (6)				
Physical activity										
Sedentary time, min · d^−1^	394.4	51.7^*^	387.4	to	401.5	379.3	48.5	371.6	to	386.9
Light physical activity, min · d^–1b^	106.8	27.3^*^	103.1	to	110.5	117.0	36.2	111.2	to	122.7
MVPA, min · d^–1b^	37.4	16.7^*^	35.1	to	39.6	46.7	21.3	43.4	to	50.1
Vigorous physical activity, min · d^–1b^	5.3	6.6	4.4	to	6.2	5.2	5.9	4.3	to	6.2
Steps per day	10 465	2 394^*^	10 138	to	10 791	11 176	2 949	10 709	to	11 642
Physical activity status										
Achieve ≥60 min · d^−1^of MVPA, *N* (% of *N*)	17 (8)					34 (22)				
Number of days with wear time ≥10 h^c^	6 (1)					6 (1)				
Wear time, h · d^−1^	12.8	0.9	12.7	to	12.9	13.0	0.9	12.9	to	13.2

*N* number of participants, *SD* standard deviation, *CI* confidence interval, *BMI* body mass index, *MVPA* moderate-to-vigorous physical activity

^*^Significantly different from boys, independent samples t-test, *P* < 0.05

^a^Categories were defined by sex- and age-specific cut-off points for BMI

^b^Values were transformed to natural logarithms before analysis

^c^Expressed as the median and interquartile range

### Body composition measurement

BC was assessed with a multi-frequency bioelectrical impedance analysis using the manufacturer’s equation. BC assessment using the InBody 720 device (Biospace Co., Ltd., Seoul, Korea) is considered highly precise for measuring BC in children [29]. The InBody 720 device measures the resistance in broadband frequencies (1–1000 kHz) and reactance in mean frequencies (5–250 kHz). All measurements were performed with alternating currents of 90 μA (1 kHz) and 400 μA (other frequencies). The measurement was performed in a standing position while the participant was barefoot and wearing light indoor clothing. To maintain examination validity, the study participants were instructed to fast for at least 4 h and maintain proper hydration for at least 24 h before the examination. One field worker performed the BC measurement on school premises during the morning hours (8:00–11:30).

### Anthropometric measurement

The standing height was measured before BC measurement using a standard procedure with an accuracy of 0.1 cm by means of Anthropometer P-375 (Trystom, Olomouc, Czech Republic). Body weight was measured using the InBody 720 instrument with an accuracy of 0.1 kg. Sex- and age-specific BMI values were used to categorize participants as underweight [30], normal weight, overweight or obese [31]. FMI was calculated by dividing the FM by height squared.

### Physical activity measurement

PA of various intensities was monitored seven consecutive days by a hip-worn accelerometer ActiGraph (ActiGraph, LLC., FL, USA), the use of which is considered sufficiently valid in children [32]. The accelerometers were given to the children immediately after completing the BC assessment. Information about the correct wearing of the accelerometer was explained before the first attachment; this information was given to the parents in written form so that they could check and correct the attachment on a regular basis. The children were further instructed to attach the accelerometer in the morning, right after waking up and to detach the accelerometer in the evening, before going to bed or whenever they came into contact with water (e.g., swimming or taking a shower).

Before testing, each accelerometer was calibrated according to the manufacturer’s recommendations. The time sampling interval was set at 60 s. Sedentary time, time spent in light PA (LPA), MVPA and vigorous PA (VPA) were calculated from raw accelerometer data (i.e. counts) using the cut-off points defined by Evenson et al. [33]. For data to be considered valid, the following two criteria were established: a minimum of 10 h of wearing time per day and at least 4 days including one weekend day of recoded PA. The mean wearing time was 12.9 ± 0.9 h∙d^−1^, and 65% of all children had 6 or 7 valid days. Two hundred and sixty-seven children were excluded from the analysis. These children did not achieve the wearing time criteria or their data could not be assessed due to technical failures during downloading or loss of the device. Therefore, the final sample consisted of 365 children. The basic PA characteristics are displayed in Table 1.

### Data analysis

All calculations were made using SPSS software, version 21 (SPSS for Windows; SPSS, Chicago, IL). The descriptive statistics of the outcome measures are presented as the mean, standard deviation and 95% confidence interval unless stated otherwise. Statistical data analysis was performed separately for girls and boys because previous studies indicated that the association between PA and BC in children was sex-specific [23, 24, 34]. All analyses were 2-tailed and were performed with the alpha value set at 0.05. Due to the skewed nature of VAT, LPA, MVPA and VPA, we used the natural logarithm in all statistical analyses.

The differences in PA and anthropometric characteristics between girls and boys were assessed using independent samples t-test after testing the homogeneity of variances with Levene’s test; to compare the categorical factors (sex, weight and PA status), Pearson’s Chi-square test was used. To compare the differences in body fatness indicators, the individuals were divided into three groups (tertiles) according to the MVPA values. The cut-off points of the tertiles were 28.8 and 41.1 min∙d^−1^ for girls and 39.8 and 51 min∙d^−1^ for boys. To assess the significance of the differences in the average values of the body fatness indicators between the tertiles, Fisher’s LSD post hoc test was used after a one-way analysis of variance. A linearity tests were applied for the initial verification of a possible associations (trend).

We examined the associations of LPA, MVPA and VPA with body fatness indicators using a linear regression analysis that was adjusted for potential confounding variables. To compare the regression coefficients across the outcome measures, we used standardized values (Z-score). Model 1 represents an explorative (unadjusted) model, which does not consider the possible influence of confounders. Model 2 was adjusted for model 1 and age. Regarding the fact that there was a strong quadratic relationship between the FM and body height for both sexes, for these parameters, model 2 was also adjusted for body height to eliminate the effect of various body heights. Model 3 was adjusted for model 2 and sedentary time to determine whether the associations between PA of different intensities and body fatness indicators were independent of sedentary time.

## Results

Of the total number of 632 children whose parents provided written consent, the final analysis included 365 (58%) children with an average age of 9.8 ± 1.3 years. The descriptive characteristics for these participants, grouped by sex, are displayed in Table 1. The mean age, body height, weight and BMI did not differ between the groups of girls and boys. Girls reported 15.1 min∙d^−1^ more sedentary time than boys (*t* = 2.85; *P* = 0.005), while boys accumulated a higher level of light PA (*t* = –3.07; *P* = 0.002), MVPA (*t* = –4.48; *P* < 0.001) and steps per day (*t* = –2.54; *P* = 0.011) than girls. We found a significant association between sex and achievement of the recommended level of MVPA per day (*χ* ^2^ = 13.87; *P* < 0.001).

Although a significant difference in body fatness indicators between the MVPA tertiles was observed only in FM% in girls (*F* = 3.09; *P* = 0.048), a typical feature of both sexes was that the values of FM, FM%, FMI and VAT were significantly higher in the first tertile compared with the third tertile (Table 2). Moreover, we confirmed a linear trend for these variables, except FM in girls, where the selected level of significance was slightly exceeded (*P*_trend_ = 0.051).

**Table 2:** Differences in body fatness indicators between differentially active children grouped by sex

	First tertile		Second tertile		Third tertile		*F*	*P*_MVPA_	*P*_trend_
	Mean	SD	Mean	SD	Mean	SD			
Girls (*N* = 209)									
Fat mass, kg	7.9	5.5^*^	6.9	4.4	6.4	3.9	1.97	0.142	0.051
Fat mass, %	20.1	8.5^*^	18.1	7.3	16.9	7.1	3.09	0.048	0.015
Fat mass index, kg · m^−2^	3.8	2.4^*^	3.3	1.9	3.1	1.8	2.37	0.096	0.035
Visceral adipose tissue, cm^2a^	37.3	27.3^*^	31.4	22.6	28.0	21.8	2.26	0.107	0.036
Boys (*N* = 156)									
Fat mass, kg	7.8	5.8^*^	6.7	5.2	5.7	3.8	2.21	0.113	0.038
Fat mass, %	18.4	8.9^*^	16.1	8.3	15.0	7.0	2.36	0.092	0.035
Fat mass index, kg · m^−2^	3.6	2.4^*^	3.1	2.3	2.8	1.7	2.12	0.123	0.044
Visceral adipose tissue, cm^2a^	42.3	32.0^*^	34.4	26.5	28.3	21.0	2.42	0.092	0.030

The cut-off points of the tertiles were 28.8 and 41.1 min · d^−1^ for girls and 39.8 and 51 min · d^−1^ for boys

*N* number of participants, *SD* standard deviation, *MVPA* moderate-to-vigorous physical activity, *P*_*MVPA*_ *P*-value for one-way analysis of variance, *P*_*trend*_ *P*-value for linearity test

^*^Significantly different from the third tertile, Fisher’s LSD post-hoc test after one-way analysis of variance, *P* < 0.05

^a^Values were transformed to natural logarithms before analysis

The raw and adjusted associations of different intensities of PA with body fatness indicators are shown in Tables 3 and 4. No significant associations were found between LPA and all body fatness indicators in either sex, even though sedentary time was taken into consideration as a covariate in the fully adjusted model. In girls, MVPA was significantly negatively associated with body fatness, represented by the FM, FM%, FMI and VAT (β = –0.46 to –0.32; all *P* < 0.05); this association strengthened after adjusting for age and body height (β = –0.51 to –0.38; all *P* < 0.05) and was independent of sedentary time. In boys, we observed a negative, non-significant association between MVPA and all body fatness indicators (β = –0.31 to –0.28; all *P* > 0.05); the association weakened after the model was adjusted for all confounding factors. Strong associations between VPA and body fatness indicators were found in boys, but there were no strong associations in girls. VPA was strongly associated with FM (β = –0.16, *P* = 0.029), FM% (β = –0.16, *P* = 0.034) and FMI (β = –0.16, *P* = 0.027) in the unadjusted model. After adjusting the model for all confounding factors, VPA was significantly associated with FM% (β = –0.15, *P* = 0.048) and FMI (β = –0.15, *P* = 0.040). Finally, the risk of collinearity was tested to avoid bias of regression models. We found a weak correlation (Pearson’s r ranging from –0.12 to 0.13) between sedentary time and time spent in PA of different intensities which could not have a profound effect on the estimated associations.

**Table 3:** Associations between physical activity of different intensities and body fatness indicators Z-scores in girls (*N* = 209)

	Fat mass (kg)			Fat mass (%)			Fat mass index (kg · m^−2^)			Visceral adipose tissue^a^ (cm^2^)		
	β^b^	(95% CI)	*P*-value	β^b^	(95% CI)	*P*-value	β^b^	(95% CI)	*P*-value	β^b^	(95% CI)	*P*-value
LPA^a^												
Model 1	−0.03	(–0.56, 0.50)	0.901	−0.06	(–0.59, 0.47)	0.817	0.10	(–0.43, 0.64)	0.699	−0.30	(–0.83, 0.23)	0.268
Model 2	0.47	(–0.03, 0.97)	0.062	0.15	(–0.40, 0.69)	0.596	0.33	(–0.21, 0.88)	0.229	−0.03	(–0.57, 0.51)	0.903
Model 3	0.41	(–0.09, 0.92)	0.107	0.07	(–0.49, 0.62)	0.812	0.27	(–0.28, 0.82)	0.332	−0.10	(–0.64, 0.45)	0.731
MVPA^a^												
Model 1	−0.37	(–0.65, –0.09)	0.010	−0.46	(–0.74, –0.18)	0.001	−0.44	(–0.72, –0.16)	0.002	−0.32	(–0.60, –0.04)	0.026
Model 2	−0.47	(–0.72, –0.22)	<0.001	−0.51	(–0.78, –0.23)	<0.001	−0.48	(–0.76, –0.21)	<0.001	−0.38	(–0.65, –0.10)	0.008
Model 3	−0.46	(–0.71, –0.21)	<0.001	−0.49	(–0.76, –0.22)	<0.001	−0.47	(–0.75, –0.20)	0.001	−0.36	(–0.64, –0.09)	0.010
VPA^a^												
Model 1	−0.04	(–0.15, 0.07)	0.508	−0.08	(–0.19, –0.04)	0.181	−0.05	(–0.16, 0.06)	0.367	−0.06	(–0.18, 0.06)	0.349
Model 2	−0.06	(–0.15, 0.04)	0.273	−0.09	(–0.20, 0.02)	0.122	−0.06	(–0.17, 0.05)	0.269	−0.07	(–0.19, 0.05)	0.228
Model 3	−0.05	(–0.15, 0.05)	0.287	−0.09	(–0.20, 0.03)	0.130	−0.06	(–0.17, 0.05)	0.282	−0.07	(–0.18, 0.05)	0.238

Model 1 – unadjusted (raw) model

Model 2 – model 2 was adjusted for age and body height

Model 3 – model 3 was adjusted for model 2 and sedentary time

*CI* confidence interval, *LPA* light physical activity, *MVPA* moderate-to-vigorous physical activity, *VPA* vigorous physical activity

^a^Values were transformed to natural logarithms before analysis

^b^β gives the regression coefficient of the standardized values (Z-score) for each variable

**Table 4:** Associations between physical activity of different intensities and body fatness indicators Z-scores in boys (*N* = 156)

	Fat mass (kg)			Fat mass (%)			Fat mass index (kg · m^−2^)			Visceral adipose tissue^a^ (cm^2^)		
	β^b^	(95% CI)	*P*-value	β^b^	(95% CI)	*P*-value	β^b^	(95% CI)	*P*-value	β^b^	(95% CI)	*P*-value
LPA^a^												
Model 1	−0.27	(–0.77, 0.23)	0.281	−0.28	(–0.77, 0.22)	0.272	−0.22	(–0.72, 0.27)	0.375	−0.29	(–0.78, 0.21)	0.260
Model 2	−0.07	(–0.55, 0.40)	0.757	−0.16	(–0.68, 0.36)	0.540	−0.09	(–0.61, 0.44)	0.747	−0.08	(–0.60, 0.43)	0.754
Model 3	−0.07	(–0.55, 0.41)	0.781	−0.16	(–0.69, 0.37)	0.544	−0.08	(–0.60, 0.45)	0.779	−0.12	(–0.64, 0.40)	0.653
MVPA^a^												
Model 1	−0.28	(–0.61, 0.06)	0.104	−0.31	(–0.64, 0.03)	0.071	−0.28	(–0.61, 0.05)	0.098	−0.30	(–0.63, 0.03)	0.078
Model 2	−0.25	(–0.55, 0.06)	0.111	−0.27	(–0.61, 0.06)	0.111	−0.24	(–0.58, 0.09)	0.158	−0.24	(–0.57, 0.09)	0.155
Model 3	−0.26	(–0.56, 0.05)	0.103	−0.28	(–0.61, 0.06)	0.109	−0.25	(–0.59, 0.09)	0.145	−0.22	(–0.55, 0.11)	0.193
VPA^a^												
Model 1	−0.16	(–0.30, –0.02)	0.029	−0.16	(–0.30, –0.01)	0.034	−0.16	(–0.30, –0.02)	0.027	−0.12	(–0.27, 0.03)	0.119
Model 2	−0.12	(–0.25, 0.01)	0.071	−0.15	(–0.29, –0.01)	0.048	−0.15	(–0.29, –0.01)	0.039	−0.10	(–0.25, 0.05)	0.175
Model 3	−0.12	(–0.25, 0.01)	0.073	−0.15	(–0.29, –0.01)	0.048	−0.15	(–0.29, –0.01)	0.040	−0.10	(–0.25, 0.04)	0.168

Model 1 – unadjusted (raw) model

Model 2 – model 2 was adjusted for age and body height

Model 3 – model 3 was adjusted for model 2 and sedentary time

*CI* confidence interval, *LPA* light physical activity, *MVPA* moderate-to-vigorous physical activity, *VPA* vigorous physical activity

^a^Values were transformed to natural logarithms before analysis

^b^β gives the regression coefficient of the standardized values (Z-score) for each variable

## Discussion

In the present study we observed a decrease in body fatness in both girls and boys represented by FM, FM%, FMI and VAT, from the first to the third tertile, which was determined according to an average MVPA level per day. A regression analysis revealed a strong negative association between objectively measured PA and all body fatness indicators. These associations were sex-specific and were independent of potential confounding factors including sedentary time. A significant association between MVPA and body fatness indicators was observed only in girls; the association strengthened after adjustment for covariates. On the contrary, VPA was strongly associated with body fatness represented by FM% and FMI in boys.

The results presented in this manuscript correspond with the conclusions of a systematic review by Jiménez-Pavón et al. [20], who compared a total of 48 studies on the associations between PA and body fatness in children. A majority of analyzed studies confirmed a strong negative association between objectively measured PA and body fatness, which seems to be influenced by a number of factors. In contrast to our results, Ekelund et al. [35] confirmed that although the accumulated amount of time spent in MVPA is related to FM, this relationship was weak because the explained variance was less than 1%. Similar conclusions were formulated by Rowlands et al. [26], who claimed that a greater level of accumulated MVPA was not associated with less FM% in either of the sexes. On the other hand, Ness et al. [24] observed strong negative associations between MVPA and total PA and FM in both sexes. Compared with girls, the associations in boys were stronger, particularly those of MVPA compared with total PA. Strong negative associations between MVPA and FM%, independent of sedentary time, were observed in Canadian children in the QUALITY cohort study [28]. There is also evidence suggested by prospective studies on the associations of PA with body fatness in children. In a recent cohort study, Ramires et al. [25] showed a protective effect of self-reported VPA on FM only in boys, which corresponds with our results. Moreover, a few prospective studies used objective monitoring of PA. For example, Riddoch et al. [27] found that a higher level of accelerometer-determined MVPA at the age of 12 was prospectively associated with lower levels of FM in early adolescence in both sexes.

Although more MVPA might be strongly associated with lower FM, it appears that this association depends on the amount of VPA, which promotes larger energy costs and seems to be an important component for preventing obesity [36]. In our study, a larger level of accumulated VPA was associated with lower body fatness in boys. For both sexes, this trend was confirmed in a study by Dencker et al. [37]. In this study, the authors observed a significant negative association between VPA and FM% in Swedish children. The association strengthened after adjusting for sex; no significant associations were found for moderate PA. Likewise, Rowlands et al. [26] observed significant correlations between total PA and VPA and FM% in both sexes.

The results presented in this study indicate that the strength of the association between PA and body fatness is also influenced by sex. We observed a significant association between MVPA and body fatness in girls, while there was a strong association for VPA in boys. The effect of sex on the association between PA and body fatness is also confirmed by Treuth et al. [23], who state that greater sedentary time was associated with greater FM% and that more time spent in light PA was associated with lower FM% in girls, which was not the case for boys. By contrast, Saelens et al. [34] described stronger associations between total PA and body fatness in boys, even for VAT, which is associated with a higher risk of medical complications than subcutaneous fat. As far as VAT is concerned, this study revealed a significant association for MVPA in girls. A typical feature of the monitored age category is an increase in body weight. In comparison with boys, the age-related increase in body weight in girls is caused particularly by a gain in FM rather than a gain in fat-free mass [38]. These results correspond with our study in which girls reported significantly more FM% (unpublished data) than boys. Thus, the differences in the association between PA and body fatness might be affected by specific development of each sex during childhood.

There are several limitations that should be considered when interpreting the findings of the present study. First, in the case of PA assessment, accelerometer epoch lengths must be considered. Just as in our study, research focusing on PA in children usually has a 60-s epoch [39]. However, a longer epoch might result in underestimation of higher-intensity PA. Therefore, a shorter epoch is recommended to obtain a more precise record of PA behavior in children and adolescents [40]. The proportion of individuals accumulating MVPA ≥60 min∙d^−1^ is relatively low in the present study (14% of the sample) compared with previously published values of Czech children and adolescents [9], which could suggest underestimation of MVPA. However, a comparison of these results is merely informative because of the different methods of determining PA (accelerometer vs. questionnaire). Second, the accelerometer data reduction criteria might also present a limitation. The minimum requirements for daily wear time (i.e., ≥10 h∙d^−1^) and valid days (at least 4 days including 1 weekend day) might have a substantial impact on sample size and could result in a selection bias [41]. Moreover, Rowlands et al. [42] reported that the level of PA in children decreases during the weekend, while the sedentary time increases. For this reason, MVPA and VPA could have been underestimated in individuals who included two weekend days (68% of the sample). Underestimation of MVPA and VPA due to a decrease in PA during the weekend affects especially less active children as they report a significant increase in sedentary time and decrease in PA of all intensities [43]. Finally, although we checked the association between PA and body fatness indicators for age, body height and sedentary time there are other confounding factors, such as maturational status, parental weight, PA, socioeconomic status, energy intake and sexual maturity. These factors should also be considered when interpreting the results.

Although scientific evidence has confirmed a long-term trend of increasing overweight and obesity prevalence in Czech children and adolescents [7–9], there is lack of studies using objective methods. The potential strength of this study is the fact that we objectively measured body fatness using a multi-frequency bioelectrical impedance analysis and PA levels using accelerometers. Thus, this study significantly contributes to our understanding of the importance of PA in the prevention of overweight and obesity among Czech children.

## Conclusions

In conclusion, we have shown a strong negative association of accelerometer-based PA with body fatness in Czech children who are 7 to 12 years of age. This association seems to depend on the sex and PA intensity. In girls, we observed a strong association between MVPA and all body fatness indicators, while in boys, we observed a significant adjusted association between VPA and body fatness indicators represented by FM% and FMI. These results support the hypothesis that higher PA levels precede increased FM gain and may help to reduce the prevalence of overweight and obesity in children. Longitudinal studies are required to confirm prospective association of PA with body fatness and to test the sex-specific benefits of various intensities of PA on body fatness in children.

## Abbreviations

BC: Body composition
FM: Fat mass
FM%: Fat mass percentage
FMI: Fat mass index
LPA: Light physical activity
MVPA: Moderate-to-vigorous physical activity
PA: Physical activity
VAT: Visceral adipose tissue
VPA: Vigorous physical activity

## References

[1] World Health Organization. Obesity and overweight, Fact sheet no. 311 (January). http://www.who.int/mediacentre/factsheets/fs311/en/. Accessed 24 Mar 2016.

[2] Stamatakis E, Wardle J, Cole TJ (2010). Childhood obesity and overweight prevalence trends in England: evidence for growing socioeconomic disparities. Int J Obes.

[3] Ogden CL, Carroll MD, Kit BK, Flegal KM (2014). Prevalence of childhood and adult obesity in the United States, 2011–2012. JAMA.

[4] Swinburn BA, Sacks G, Hall KD, McPherson K, Finegood DT, Moodie ML (2011). The global obesity pandemic: shaped by global drivers and local environments. Lancet.

[5] Lobstein T, Jackson-Leach R (2016). Planning for the worst: estimates of obesity and comorbidities in school-age children in 2025. Pediatr Obes.

[6] Ng M, Fleming T, Robinson M, Thomson B, Graetz N, Margono C (2014). Global, regional, and national prevalence of overweight and obesity in children and adults during 1980–2013: a systematic analysis for the Global Burden of Disease Study 2013. Lancet.

[7] Kunešová M, Vignerová J, Pařízková J, Procházka B, Braunerová R, Riedlová J (2011). Long-term changes in prevalence of overweight and obesity in Czech 7-year-old children: evaluation of different cut-off criteria of childhood obesity. Obes Rev.

[8] Vignerová J, Humeníková L, Paulová M, Riedlová J (2008). Prevalence of overweight, obesity and low weight in the Czech child population up to 18 years of age in the last 50 years. J Public Health.

[9] Sigmundová D, Sigmund E, Hamřík Z, Kalman M (2014). Trends of overweight and obesity, physical activity and sedentary behaviour in Czech schoolchildren: HBSC study. Eur J Public Health.

[10] Reilly JJ, Kelly J (2011). Long-term impact of overweight and obesity in childhood and adolescence on morbidity and premature mortality in adulthood: systematic review. Int J Obes.

[11] Gába A, Dygrýn J, Mitáš J, Jakubec L, Frömel K (2016). Effect of accelerometer cut-off points on the recommended level of physical activity for obesity prevention in children. PLoS One.

[12] Waters E, de Silva-Sanigorski A, Hall BJ, Brown T, Campbell KJ, Gao Y, et al. Interventions for preventing obesity in children. Cochrane Database Syst Rev. 2011; CD001871. doi:10.1002/14651858.CD001871.pub3. 10.1002/14651858.CD001871.pub3 22161367

[13] World Health Organization. Global recommendations on physical activity for health [brochure]. http://www.who.int/dietphysicalactivity/factsheet_recommendations/en/. Accessed 24 March 2016.

[14] U. S. Department of Health and Human Services. 2008 Physical activity guidelines for Americans be active, healthy, and happy! [brochure]. http://health.gov/paguidelines/guidelines/. Accessed 24 Mar 2016.

[15] Department of Health and Ageing. Make your move – Sit less – Be active for life! (Children) [brochure]. http://www.health.gov.au/internet/main/publishing.nsf/content/health-pubhlth-strateg-phys-act-guidelines. Accessed 24 Mar 2016.

[16] Tremblay MS, Warburton DE, Janssen I, Paterson DH, Latimer AE, Rhodes RE (2011). New Canadian physical activity guidelines. Appl Physiol Nutr Metab.

[17] Hallal PC, Andersen LB, Bull FC, Guthold R, Haskell W, Ekelund U (2012). Global physical activity levels: surveillance progress, pitfalls, and prospects. Lancet.

[18] Nader PR, Bradley RH, Houts RM, McRitchie SL, O’Brien M (2008). Moderate-to-vigorous physical activity from ages 9 to 15 years. JAMA.

[19] Troiano RP, Berrigan D, Dodd KW, Masse LC, Tilert T, McDowell M (2008). Physical activity in the United States measured by accelerometer. Med Sci Sports Exerc.

[20] Jiménez-Pavón D, Kelly J, Reilly JJ (2010). Associations between objectively measured habitual physical activity and adiposity in children and adolescents: Systematic review. Int J Pediatr Obes.

[21] Ramires VV, Dumith SC, Goncalves H (2015). Longitudinal association between physical activity and body fat during adolescence: a systematic review. J Phys Act Health.

[22] Katzmarzyk PT, Shen W, Baxter-Jones A, Bell JD, Butte NF, Demerath EW (2012). Adiposity in children and adolescents: correlates and clinical consequences of fat stored in specific body depots. Pediatr Obes.

[23] Treuth MS, Hou N, Young DR, Maynard LM (2005). Accelerometry-measured activity or sedentary time and overweight in rural boys and girls. Obes Res.

[24] Ness AR, Leary SD, Mattocks C, Blair SN, Reilly JJ, Wells J (2007). Objectively measured physical activity and fat mass in a large cohort of children. PLoS Med.

[25] Ramires VV, Dumith SC, Wehrmeister FC, Hallal PC, Menezes AMB, Gonçalves H (2016). Physical activity throughout adolescence and body composition at 18 years: 1993 Pelotas (Brazil) birth cohort study. Int J Behav Nutr Phys Act.

[26] Rowlands AV, Eston RG, Powell SM (2006). Total physical activity, activity intensity and body fat in 8-11-year-old boys and girls. J Exerc Sci Fit.

[27] Riddoch CJ, Leary SD, Ness AR, Blair SN, Deere K, Mattocks C (2009). Prospective associations between objective measures of physical activity and fat mass in 12-14 year old children: the Avon Longitudinal Study of Parents and Children (ALSPAC). BMJ.

[28] Chaput JP, Lambert M, Mathieu ME, Tremblay MS, O’ Loughlin J, Tremblay A (2012). Physical activity vs. sedentary time: independent associations with adiposity in children. Pediatr Obes.

[29] Lim JS, Hwang JS, Lee JA, Kim DH, Park KD, Jeong JS (2009). Cross-calibration of multi-frequency bioelectrical impedance analysis with eight-point tactile electrodes and dual-energy X-ray absorptiometry for assessment of body composition in healthy children aged 6–18 years. Pediatr Int.

[30] Cole TJ, Flegal KM, Nicholls D, Jackson AA (2007). Body mass index cut offs to define thinness in children and adolescents: international survey. BMJ.

[31] Cole TJ, Bellizzi MC, Flegal KM, Dietz WH (2000). Establishing a standard definition for child overweight and obesity worldwide: international survey. BMJ.

[32] de Vries SI, Bakker I, Hopman-Rock M, Hirasing RA, van Mechelen W (2006). Clinimetric review of motion sensors in children and adolescents. J Clin Epidemiol.

[33] Evenson KR, Catellier DJ, Gill K, Ondrak KS, McMurray RG (2008). Calibration of two objective measures of physical activity for children. J Sports Sci.

[34] Saelens BE, Seeley RJ, van Schaick K, Donnelly LF, O’Brien KJ (2007). Visceral abdominal fat is correlated with whole-body fat and physical activity among 8-y-old children at risk of obesity. Am J Clin Nutr.

[35] Ekelund U, Sardinha LB, Anderssen SA, Harro M, Franks PW, Brage S (2004). Associations between objectively assessed physical activity and indicators of body fatness in 9- to 10-y-old European children: a population-based study from 4 distinct regions in Europe (the European Youth Heart Study). Am J Clin Nutr.

[36] Laguna M, Ruiz JR, Lara MT, Aznar S (2013). Recommended levels of physical activity to avoid adiposity in Spanish children. Pediatr Obes.

[37] Dencker M, Thorsson O, Karlsson MK, Lindén C, Eiberg S, Wollmer P (2006). Daily physical activity related to body fat in children aged 8–11 years. J Pediatr.

[38] McCarthy HD, Cole TJ, Fry T, Jebb SA, Prentice AM (2006). Body fat reference curves for children. Int J Obes.

[39] Cain KL, Sallis JF, Conway TL, Van Dyck D, Calhoon L (2013). Using accelerometers in youth physical activity studies: a review of methods. J Phys Act Health.

[40] Edwardson CL, Gorely T (2010). Epoch length and its effect on physical activity intensity. Med Sci Sports Exerc.

[41] Toftager M, Kristensen PL, Oliver M, Duncan S, Christiansen LB, Boyle E (2013). Accelerometer data reduction in adolescents: effects on sample retention and bias. Int J Behav Nutr Phys Act.

[42] Rowlands AV, Pilgrim EL, Eston RG (2008). Patterns of habitual activity across weekdays and weekend days in 9–11-year-old children. Prev Med.

[43] Fairclough SJ, Boddy LM, Mackintosh KA, Valencia-Peris A, Ramirez-Rico E (2015). Weekday and weekend sedentary time and physical activity in differentially active children. J Sci Med Sport.

